# Establishing the core elements of a frailty at the front door model of care using a modified real-time Delphi technique

**DOI:** 10.1186/s12873-023-00893-9

**Published:** 2023-10-20

**Authors:** Íde O’Shaughnessy, Christine Fitzgerald, Aoife Whiston, Patrick Harnett, Helen Whitty, Des Mulligan, Marian Mullaney, Catherine Devaney, Deirdre Lang, Jennifer Hardimann, Brian Condon, Christina Hayes, Alison Holmes, Louise Barry, Claire McCormack, Megan Bounds, Katie Robinson, Margaret O’Connor, Damien Ryan, Denys Shchetkovsky, Fiona Steed, Leonora Carey, Emer Ahern, Rose Galvin

**Affiliations:** 1 School of Allied Health, Faculty of Education and Health Sciences, Ageing Research Centre, Health Research Institute, University of Limerick, Limerick, Ireland; 2 Clinical Design and Innovation, Health Service Executive, National Clinical Programme for Older People, Dublin, Ireland; 3 Department of Nursing and Midwifery, University of Limerick, Limerick, Ireland; 4 School of Medicine, Faculty of Education and Health Sciences, University of Limerick, Limerick, Ireland; 5 College of Medicine, University of Arizona, Tucson, USA; 6 Department of Ageing and Therapeutics, University Hospital Limerick, Dooradoyle, Limerick, Limerick, Ireland; 7 Emergency Department, Limerick EM Education Research Training (ALERT), University Hospital Limerick, Dooradoyle, Limerick, Ireland; 8 Department of Health, Baggot Street, Dublin, Ireland; 9 Department of Occupational Therapy, University Hospital Limerick, Dooradoyle, Limerick, Ireland

**Keywords:** Older adults, Emergency department, Frailty at the front door, Consensus, Delphi technique

## Abstract

**Background:**

Innovations in models of care for older adults living with frailty presenting to the emergency department (ED) have become a key priority for clinicians, researchers and policymakers due to the deleterious outcomes older adults experience due to prolonged exposure to such an environment. This study aimed to develop a set of expert consensus-based statements underpinning operational design, outcome measurement and evaluation of a Frailty at the Front Door (FFD) model of care for older adults within an Irish context.

**Methods:**

A modified real-time Delphi method was used. Facilitation of World Café focus groups with an expert panel of 86 members and seperate advisory groups with a Public and Patient Involvement panel of older adults and members of the Irish Association of Emergency Medicine generated a series of statements on the core elements of the FFD model of care. Statements were analysed thematically and incorporated into a real-time Delphi survey, which was emailed to members of the expert panel. Members were asked to rank 70 statements across nine domains using a 9-point Likert scale. Consensus criteria were defined a priori and guided by previous research using 9-point rating scales.

**Results:**

Fifty members responded to the survey representing an overall response rate of 58%. Following analyses of the survey responses, the research team reviewed statements for content overlap and refined a final list of statements across the following domains: aims and objectives of the FFD model of care; target population; screening and assessment; interventions; technology; integration of care; evaluation and metrics; and research.

**Conclusion:**

Development of a consensus derived FFD model of care represents an important step in generating national standards, implementation of a service model as intended and enhances opportunities for scientific impact. Future research should focus on the development of a core outcome set for studies involving older adults in the ED.

**Supplementary Information:**

The online version contains supplementary material available at 10.1186/s12873-023-00893-9.

## Background

The significant growth in Emergency Department (ED) attendances is an international public health issue posing a major risk to population health [1]. ED crowding affects the quality and safety of patient care and the association with a higher risk of adverse outcomes [2, 3] and increased mortality rates are well-documented [4, 5]. As conceptualised within the Input-Throughput-Output model [6], the causes and consequences of ED crowding are complex and multifaceted [7]. The most potent input factor driving ED crowding is population ageing [8]. Although frailty is not synonymous with age, international evidence reflects a disproportionate level of ED attendance amongst older adults aged ≥ 65 relative to the demographic trajectory [9]. The ED has long been recognised as a challenging environment to deliver effective care to older adults living with frailty [10]. Emergency Medicine (EM) has traditionally emphasised time-based targets and protocolised care for disease-specific presentations like stroke, trauma and sepsis [11, 12]. However, the multi-complexity of older adults’ presentations may pose challenges to emergency care [13] and investment in additional time and resources are indicated to complete a holistic assessment across multiple domains [14]. Innovations in models of care for older adults living with frailty presenting to the ED have therefore become a key priority for clinicians, researchers and policymakers [11].

Unifying all evidence-based changes to enhance health services delivery for older adults living with frailty is comprehensive geriatric assessment (CGA) [15], which is defined as a ‘multidimensional diagnostic and therapeutic process focussed on determining a frail older person’s medical, psychological and functional capability in order to develop a coordinated and integrated plan for treatment and follow-up’ [16]. CGA has been shown to improve a range of outcomes in hospitalised older adults [17]; however, despite serving as the main portal of entry to inpatient care, limited high quality evidence exists to support the effectiveness of CGA in the ED [18]. A recent scoping review of the literature synthesised the evidence on models of care for older adults living with frailty presenting to the ED [19]. Findings showed that the 13 studies included in the review were heterogeneous in nature with respect to use of screening and assessment tools to identity frailty and support decision-making, team composition and outcomes reported. The authors concluded that critical analysis of existing policies, guidelines and models of care is required before implementing new service models for older adults living with frailty in the ED.

In Ireland, substantial investment has been provided by the national Health Service Executive (HSE), in the form of a Frailty at the Front Door (FFD) model of care. The FFD model of care is underpinned by the principles of CGA whereby an interdisciplinary team of healthcare professionals proactively identify complex and multifactorial needs in older adults through completion of a holistic assessment and initiation of a tailored treatment plan. FFD teams reflect a component of an integrated model of care developed by the National Clinical Programme for Older People (NCPOP) and emphasise timely access to CGA and development of end-to-end pathways that describe cohesive primary, secondary, and acute care services for older adults living with frailty [20]. However, while scale up of the FFD model of care is at an advanced stage, variations have emerged in terms of design, fidelity of approach, implementation and evaluation of impact. Consequently, the lack of homogeneity, underpinned by evidence, has implications for the optimum operating model and stakeholder expectations.

Our study aims to develop a set of expert consensus-based statements on the core elements underpinning the operational delivery, outcome measurement and evaluation of the FFD model of care for older adults within an Irish context.

## Methods

The Delphi technique is a well-established method used to achieve consensus among experts on a particular topic in a systematic manner [21, 22]. There are many variations of the classic Delphi method, with the option for researchers to modify the approach to reflect research aims [23]. The current study utilised a modified real-time Delphi method to develop consensus on the core elements of the FFD model of care [24]. The real-time Delphi method comprises a round-less approach, continuously calculating and updating aggregated responses in real-time until the end of study timeframe [25]. Iteration can be incorporated by email prompts providing participants with the opportunity to re-visit and re-respond to survey questions based on group responses and possible changes in consensus [26].

### Expert panel selection and recruitment

A purposive sampling strategy was used to form a national panel of FFD experts. Several definitions of ‘expert’ exist in the literature ranging from someone who has knowledge about a specific topic, considered a specialist in the field, to an informed individual [27]. In the context of this study, an expert was defined as a senior clinician working on a FFD team or a manager of the FFD service model. All 24 FFD teams nationally were contacted through NCPOP and each site was asked to nominate between 3 and 5 expert members to participate in the process of consensus building. An overview of the range of healthcare professionals participating in the expert panel (N = 86) is outlined in Table 1. To incorporate an experiential element to the study, a Public and Patient Involvement (PPI) advisory group of older adults and those important to them were also invited to contribute as research partners. The PPI advisory group provided insights and guidance to the process, ensuring meaningful representation of a lived-experience perspective when identifying priorities of the FFD model of care [28, 29]. The PPI panel were recruited from a subsample of older adults and those important to them (N = 6) who had recent experience of CGA in the ED as part of a prospective cohort study [30].

### Preparation for the Delphi process

This qualitative study employed a participatory design approach to data collection when exploring the FFD model of care for older adults [31]. The first element of the Delphi preparatory process was the facilitation of World Café focus groups (http://www.theworldcafe.com/key-concepts-resources/world-cafe-method/). Focus groups were conducted with members of the expert panel (N = 86), enabling collective knowledge and sharing of rich and diverse insights in an inclusive environment [32, 33]. The World Café procedure adhered to in this study is presented in Additional file 1. For the purposes of focus group facilitation, the FFD model of care was categorised into nine domains across two pillars: (1) operational delivery and (2) measurement. Members of the research team (RG, PH, EA, DM, HW, MM, DL, CD, JH, BC, CH, AH, LB, MB, and CMcC) facilitated focus groups and invited expert panel members to brainstorm and discuss specific aspects of each pillar through use of broad questions. An independent facilitator acted as a notetaker with 20–25 members in each group. World Café group notes were transcribed and NVivo 12 Pro was utilised to assist with data management. A reflexive thematic approach to analysis was employed using the six steps of thematic analysis as described by Braun and Clarke [34]. This approach was used as a guide to facilitate the generation of knowledge embedded in the insights and experiences of the multi-level participant group. Phase 1 included familiarisation with study data, while phase 2 involved more in-depth engagement with data and extraction of initial codes. Phases 3 and 4 resulted in the generation of initial themes and refinement of these themes by reviewing the link between the themes and the original dataset. In phases 5 and 6, themes were agreed following review and consultation between study investigators. These themes mapped to the nine focus areas in relation to aims and objectives of the FFD model of care; target population; screening and assessment; interventions; technology; integration of care; evaluation and metrics; and research.

Following the World Café, the PPI advisory group comprising a panel of four older adults and two caregivers was facilitated by two members of the research team (ÍO’S & CF). The PPI advisory group ensured older adults and those important to them perceptions and experiences of healthcare utilisation specific to the FFD model of care were explored and represented when ascertaining core elements of the service model. Further information on the structure and format of the PPI advisory group is outlined in Additional file 2.

Upon completion of both fora outlined above, two members of the research team (ÍO’S & CF) met with members of the Irish Association of Emergency Medicine (IAEM) at their annual scientific meeting, pre-conference workshop (https://iaem.ie/professional/asm2022/). The purpose of this workshop was to ensure representation of the EM voice in the Delphi process, when exploring key aspects of the FFD model and to corroborate data saturation with respect to generation of themes.

**Table 1:** Overview of healthcare professionals participating in expert panel

Profession	N
Registered Nurse	26
Physician (N = 16 Consultant Geriatrician, N = 1 Emergency Medicine Consultant)	17
Physiotherapist	17
Occupational Therapist	17
Speech and Language Therapist	4
Pharmacist	2
Dietitian	2
Social Worker	1

### The Delphi process

#### Survey design

The real-time Delphi survey was developed using the Qualtrics online survey tool and emailed to expert panel members who had previously taken part in the World Café focus groups. Statements incorporated into the survey were developed on the basis of themes that were produced from the World Café focus groups, PPI advisory group and IAEM pre-conference workshop. Survey participants were asked to rank 70 statements using a 9-point Likert scale (1 = not important to 9 = very important) across the following domains: aims and objectives of the FFD model of care; target population; screening and assessment; interventions; technology; integration of care; evaluation and metrics; and research. A full list of statements contained in the survey are outlined in Additional file 3. Space was made available at the end of the survey for free text comments.

Participation was asynchronous and members were able to re-visit the survey portal, view other members’ responses in real-time and re-rank their responses at any time point between 14th November and 19th December 2022 (a total of 36 days). Reminder emails were sent to all members at week two, four and five after initial contact.

#### Data analysis

Survey responses were analysed using JASP statistical software. Descriptive and frequency statistics were calculated for each scale item within each domain. Consensus criteria for the 9-point Likert scale items were defined a priori and based on previous research [35]. Data analysis focused on examining each statement against the following consensus criteria: (1) ≥ 70% of the sample ranked the item in the 7–9 range, (2) mean item rank in the 7–9 range, and (3) median item rank in the 7–9 range with an inter-quartile range (IQR) < 3. Final consensus criteria were defined by applying the strictest of the three criteria outlined above i.e. median 7–9, IQR < 3. Descriptive and frequency statistics were then developed into tabular form.

## Results

Fifty members of the expert panel responded and completed the real-time Delphi survey representing an overall response rate of 58%. Expert panel members from each of the eight professions completed the survey with members from physiotherapy (26%), nursing (22%), and consultant physicians (22%) representing the highest respondents. The first list of statements, based on the three criteria are outlined in Additional file 4. Following analyses of the survey responses, members of the research team reviewed statements for content overlap and refined a final list of statements against the strictest consensus criteria i.e. median 7–9, IQR < 3. The final list are reported below and presented in tabular form in Additional file 5.

### Aims of the FFD model of care

Consensus on statements that described the aims of the FFD model of care included to improve the experience and outcomes of older adults living with frailty who present to the ED and to promote the age-attuning of the ED environment through bespoke pathways, processes and an interdisciplinary approach to care.

### Objectives

Consensus on statements that described the objectives of the FFD model of care included to embed CGA in the ED through early assessment and intervention of medical, functional, cognitive, and psychosocial abilities and to facilitate timely and supported patient discharge from the ED through initiation of referrals to appropriate community and inpatient services. Reduction in length of hospital stay of older adults living with frailty admitted to hospital following ED attendance also reached consensus. Reduction in incidence of nursing home admission did not research consensus.

### Target population

Consensus on statements that described the target population included older adults identified as frail with multiple complex co-morbidities. Statements that did not reach consensus included use of the ≥ 65 year age criterion and results of frailty screening to assist in refining the target population.

### Screening and assessment

Consensus on statements that described the screening and assessment process included commencement of CGA to incorporate a standardised biopsychosocial assessment of frailty, co-morbidity, polypharmacy, cognition, function and mobility, continence, nutrition, psychological and social status and use of an interdisciplinary assessment proforma as the basis to inform the intervention plan.

### Interventions

Consensus on statements that described core interventions delivered by FFD teams included provision of information to older adults and those important to them on the outcome of CGA and ED discharge plan and completion of timely handover to community based or inpatient services following ED discharge. Completion of medication reconciliation, provision of education on delirium risk reduction strategies, self-management strategies and nutritional advice, as appropriate, all reached consensus. Statements that did not reach consensus included provision of in-reach interventions to inpatient wards following admission to hospital.

### Role of technology

Consensus on statements that described the role of technology to support FFD teams included a requirement for greater information and communication technology (ICT) resources, development of electronic referral pathways and a shared e-proforma across primary and secondary care services. The role of current ICT systems effectively supporting the FFD model of care did not reach consensus.

### Integration of care

Consensus on statements that described elements to support the integration of care included development of shared protocols for the FFD model of care, standardisation of core aims, objectives and team composition, structured clinical governance regarding initiation/completion of CGA in the ED and decision making related to the CGA generated management plan. Statements that did not reach consensus included development and implementation of a national assessment proforma by FFD teams and structured operational governance regarding onward referral to community based integrated care services.

### Evaluation and metrics

Consensus on statements that described evaluation and metrics of FFD impact included the importance of measurement and evaluation of patient experience and outcomes as well as staff experience. A requirement for greater resources and training to enhance understanding and value of FFD evaluation, an UpToDate feedback system for FFD teams reporting to local governance structures and inclusion of a measure of patient experience, clinical and process outcomes as part of the evaluation process all reached consensus. Statements that did not reach consensus included reporting of quarterly or biannual FFD metrics to the HSE Acute Hospitals Division and reporting of process outcomes only as part of the evaluation process.

### Research

Consensus on statements that described research priorities included research as a key component of the FFD model of care, alignment of the FFD research agenda with the NCPOP research strategy and greater efforts are required to enhance understanding and value of research. Statements that did not reach consensus included research is valued and seen as a priority by FFD teams and postgraduate education specific to care of the older adult is a requisite for FFD team members.

## Discussion

### Summary

Through a modified real-time Delphi process, a national panel of experts reached consensus on the core elements of a FFD model of care within an Irish context. The final list of statements reflects the desired elements and standards as endorsed by the NCPOP and provides clinicians with a guiding framework to ensure homogeneity with respect to implementation and evaluation of the service model.

Older adults in the ED are clinically heterogeneous; therefore, identification of a target population for individualised assessment and interventions by FFD teams can pose challenges. While a plethora of international evidence exists to support the diagnostic and predictive accuracy of a number of frailty screening tools in the ED [36, 37], no single tool is recommended by experts to screen for frailty in the ED [38]. A recent prospective cohort study examined the predictive ability of commonly used ED frailty screening tools including the Identification of Seniors at Risk (ISAR), Clinical Frailty Scale (CFS), PRISMA-7 and InterRAI-ED. Findings demonstrated that older adults who screened positive for frailty were at significantly increased risk of adverse outcomes including ED re-attendance, hospital re-admission, functional decline, nursing home admission and death at 30 days and 6 months, regardless of screening tool used [39]. Accordingly, our consensus-based statements in relation to the population of interest included older adults identified as frail, without a specific focus on a single frailty screening tool.

Our consensus-based statements focused on biopsychosocial assessment and intervention domains as part of CGA in the ED as well as mechanisms to support an integrated and longitudinal approach to care. Given the high rates of adverse outcomes experienced by older adults following ED attendance [39–42], evidence-based interventions to support care transitions from the ED are a key priority for researchers and policymakers. A recent review of reviews (15 reviews describing 83 primary studies), which summarised evidence on interventions to improve outcomes for older adults attending the ED, revealed no individual intervention was found to be more beneficial, but interventions initiated in the ED and continued into other settings resulted in more favourable patient and process outcomes [43]. The FFD model of care represents an integrated and coordinated approach to healthcare delivery between primary and secondary care services; homogeneity in implementation of the service model will enable robust reporting and evaluation of efficacy and effectiveness.

Quality of emergency care for older adults has historically been reported using process metrics, such as length of ED stay and early re-attendance rate [44]. While useful for managers, these metrics may not capture what older adults consider meaningful. Application of patient-reported outcomes and experience measures are increasingly recognised as valid approaches to measure the quality and impact of care by clinicians, funders and policymakers [45]. In keeping with this paradigm shift to outcome measurement, our consensus-based statements reflect inclusion of measures of patient experience, clinical and process outcomes as part of the evaluation process.

The majority of statements that did not reach consensus related to the domain of research. Findings from the survey revealed that while members of the expert panel considered research to be a key component of the FFD model of care, it was not valued or seen as a priority. Given that limited high quality evidence currently exists to support the effectiveness of CGA in the ED [18, 46], development of this consensus-based FFD model of care represents an important step in generating national standards for the optimum operating model thereby increasing opportunities for scientific impact.

### Areas for future research and practice

Appraisal of older adults’ experiences and outcomes from emergency care are central tenets of the FFD model of care. Our consensus-based statements pertaining to the aims and objectives of the FFD model of care draw parallels with findings from a systematic review, which focused on evaluating the expectations and preferred outcomes from ED care among older adults [47]. However, despite a proliferation of intervention studies involving older adults in the ED, significant heterogeneity exists with respect to outcome measurement and use of validated tools [48, 49]. This outcome heterogeneity has implications for reviewing research evidence and for generating policy recommendations. Therefore, there is a need for a robustly developed core outcome set for studies involving older adults in the ED to enhance transparency and availability of comparable data nationally and internationally, and to ensure outcomes measured align with what matters to older adults.

Our consensus-based FFD model of care provides clinicians with an overarching framework that can be locally adapted and expands on the principles of CGA in emergency care settings. Development of a CGA generated management plan is a core element of the FFD model of care. The consolidated ‘5Ms’ conceptual framework is grounded in the same principles and considers each older adult holistically in terms of mobility, mentation, medication, multi-complexity and what matters most [50, 51]. Application of this framework has the potential to assist clinicians with operationalising the core elements of the FFD model of care. In keeping with its focus on cultivating an age-friendly health system [52], use of this framework facilitates the age-attuning of the ED and is therefore important for clinicians to consider when addressing the complex and interrelated needs of older adults both in the ED and across the continuum of care.

### Strengths and limitations

The strengths of this study lie in its robust methodological design and extensive stakeholder engagement. Formation of an expert panel, which was representative of FFD teams nationally augments external validity and generalisability to other healthcare systems. Involvement of the PPI advisory group of older adults and those important to them provided an important viewpoint in terms of how best to represent their priorities and preferences thereby ensuring the FFD model of care remains person-centred.

A potential limitation of the Delphi technique is researcher influence on the formulation of the statements for inclusion in the survey. However, to minimise this risk we based our statements on themes that emerged from the World Café focus groups, PPI advisory group, and IAEM workshop. Use of the real-time Delphi process mitigated against a prolonged study timeframe and potential high attrition rates, which are limitations of the classic Delphi technique. Another general limitation of the Delphi technique is the choice of consensus criterion can be arbitrary and subject to bias. To reduce risk of bias, we combined three recommended consensus thresholds covering distinct criterion: proportions within restricted ranges (≥ 70% scoring 7–9), central tendency within specific ranges (mean 7–9) and a decrease in variance (median 7–9, IQR < 3).

## Conclusion

Innovations in models of care for older adults living with frailty presenting to the ED are a key priority for clinicians, researchers, and policymakers. Through a modified real-time Delphi process, a national panel of experts reached consensus on the core elements underpinning operational design, outcome measurement and evaluation of the FFD model of care within an Irish context. Development of a consensus-derived FFD model of care represents an important step in generating national standards, implementation of a service model as intended and enhances opportunities for scientific impact. Future research should focus on the development of a core outcome set for studies involving older adults in the ED.

### Electronic supplementary material

Below is the link to the electronic supplementary material.

Supplementary Material 1

## Data Availability

All deidentified data files and data dictionary, and no analytic code are available as of 07/2023/05 at the Open Science Framework (OSF) repository at the following link: OSF | Frailty at the Front Door.

## Abbreviations

ED: Emergency Department
EM: Emergency Medicine
CGA: Comprehensive Geriatric Assessment
FFD: Frailty at the Front Door
HSE: Health Service Executive
NCPOP: National Clinical Programme for Older People
PPI: Patient and Public Involvement
IAEM: Irish Association of Emergency Medicine
IQR: Inter-quartile Range
CST OP: Community Specialist Team Older People
ICT: Information and Communication Technologies
ISAR: Identification of Seniors at Risk
CFS: Clinical Frailty Scale
