# Supplementary material for: Establishing the core elements of a frailty at the front door model of care using a modified real-time Delphi technique
Source: BMC Emerg Med. 2023 Oct 20;23:123. doi: 10.1186/s12873-023-00893-9 (PMC10588204; doi:10.1186/s12873-023-00893-9)
Supplement: Supplementary file 1 — Supplementary Material 1 [file 12873_2023_893_MOESM1_ESM.docx]

**Supplementary material**

**Additional file 1:** World café procedure

**Additional file 2:** Patient and public involvement advisory group

**Additional file 3:** Consensus statements contained in Delphi survey

**Additional file 4:** List of statements based on three consensus criteria

**Additional file 5:** Final list of statements based on strictest consensus criteria

**Additional file 1: World Café procedure**

The World Café principles and guidelines emphasise the importance of creating a hospitable environment (i.e. a café-style ambience) where individual and collective knowledge and ideas can be shared. The facilitators set the context to the event by describing the aim of the meeting and provided an overview of the Frailty at the Front Door (FFD) model of care. The venue of the event was set up with breakout sessions, which focused on specific domains of the FFD model of care. Facilitators discussed the principles and ground rules for participation before inviting participants to brainstorm and discuss specific aspects of each domain through use of broad questions. Individual worksheets were also provided, which enabled less confident participants to write down their contribution and thoughts regarding the core underpinnings of the FFD domain under discussion.

Each domain was discussed for 30-45 minutes and participants rotated across breakout sessions with approximately 20 participants per session. An independent notetaker not linked to the FFD service model recorded group discussions. After all domains were discussed across breakout sessions, the World Café facilitators summarised and shared knowledge from all the discussions with the full participant group using a visual summary on a flipchart. All participants had the option of commenting on the visual summary ensuring co-analysis of responses.

**Additional file 2: Patient and public involvement advisory group**

An advisory group, which involved consultation between members of the research team and a Public and Patient Involvement (PPI) stakeholder panel of older adults and family caregivers who had recent experience of Comprehensive Geriatric Assessment (CGA) in the emergency department (ED) (O’Shaughnessy et al., 2022) was facilitated. The PPI group provided insights and guidance to the process, ensuring meaningful representation of a lived-experience perspective when identifying priorities of the FFD model of care. When preliminary analysis and initial theme development were complete, a 2-hour long meeting was scheduled with six older adults and family caregivers of the PPI panel. The meeting was facilitated by two members of the research team and involved a presentation in lay language of the research process and preliminary themes and consensus statements followed by a facilitated discussion on how the model of care aligns with what they consider meaningful. There was broad agreement that the researchers’ interpretation and presentation of themes was in keeping with older adults and family caregivers’ previous experiences of CGA the ED and priorities of the FFD model of care.

**Additional file 3: Consensus statements contained in Delphi survey**

1. **Aim of Frailty at the Front Door model**
2. Reduce length of stay of older adults in the emergency department (ED)
3. Screen for presence and risk of frailty among older adults who present to the ED
4. Improve the experience and outcomes of older adults living with frailty who present to the ED
5. Enable early identification and assertive case management of older adults living with frailty to ensure appropriate plan of care
6. Promote the age attuning of the ED to meet the needs of older adults living with frailty through bespoke pathways, processes and an interdisciplinary approach to care

**2.** **Objectives of Frailty at the Front Door model**

1. Embed Comprehensive Geriatric Assessment (CGA) in the ED through early assessment and intervention of medical, functional, cognitive, and psychosocial abilities
2. Reduce the time from arrival to the ED to initial medical assessment and work-up for frail older adults
3. Facilitate timely and supported patient discharge from the ED through initiation of referrals to community-based integrated care services
4. Reduce the incidence of admission to hospital among older adults living with frailty who present to the ED
5. Actively support patient advocacy ensuring older adults living with frailty are admitted to appropriate in-patient setting
6. Reduce the length of stay for older adults living with frailty admitted to hospital following ED attendance
7. Reduce incidence of long-term care/nursing home admission following ED attendance
8. Promote a culture of proactive case management older adults living with frailty in the ED, ensuring clear communication on the outcome of assessments
9. Increase awareness of frailty and its presentations in the ED

**3. Target population**

1. Older adults who present to the ED with a fall as their index complaint
2. Older adults who present to the ED with mobility or functional deficits
3. Older adults on multiple medications perceived as vulnerable to side effects
4. Older adults with cognitive impairment/delirium or a known diagnosis of dementia
5. Older adults who have multiple complex co-morbidities indicative of frailty
6. All older adults aged ≥75 years who present to the ED during operational hours of the service
7. All older adults aged ≥65 years who present to the ED during operational hours of the service
8. All older adults who are identified as frail regardless of triage category
9. FFD team should use the results of frailty screening to assist with refining their target population for assessment and intervention in the ED
10. Incremental ‘assessment’ from universal use of screening by ED team members to full assessment by FFD teams
11. FFD team should increase the operational hours of the service to ensure optimised and consistent provision of care
12. FFD team should operationalise a triage system and assessment area within the ED that is dedicated to frail older adults

**4. Screening and assessment**

1. Screening for frailty should commence at triage and a positive frailty screen should signal the need for review by the FFD team
2. FFD and ED team members should commence early assessment after illness acuity has been determined at triage
3. FFD team should commence a CGA to incorporate a standardised biopsychosocial assessment of frailty, co-morbidity, polypharmacy, cognition, function and mobility, continence, nutrition, psychological and social status
4. FFD team should use an interdisciplinary assessment proforma as the basis to inform the intervention plan

**5. Interventions**

1. Assertive case management within the ED
2. Provide patient and family information on the outcome of CGA and ED discharge plan
3. Complete timely handover to Community Intervention Team/ Community Specialist Teams for Older Persons (CST OP)/Community Health Network following ED discharge
4. Provide in-reach to in-patient wards following admission to hospital from the ED
5. Provide patient and family with information on resources and supports available in the community
6. Prescribe enabling equipment to address unresolved functional deficits, as appropriate
7. Conduct medicine reconciliation, as appropriate
8. Provide home exercise/activity programmes, as appropriate
9. Provide family and/or caregiver education on delirium risk reduction strategies, as appropriate
10. Educate the patient and family on self-management strategies, as appropriate
11. Provide nutritional advice, as appropriate
12. Refer patients with continence issues to follow-up services in the community
13. Engage in person-centred care planning inclusive of initiation of out-of-hospital referral pathways such as CST OP

**6. Use of technology**

1. Current technology effectively supports FFD
2. Greater ICT resources are required to fully support FFD
3. Specific resources to support FFD should include electronic referral pathways to CST OP
4. Specific resources to support FFD should include a shared e-proforma across primary and secondary care

**7. Integration of care**

1. The development of shared protocols for the FFD model of care nationally would benefit service development and patient care
2. A national assessment proforma should be developed and implemented for use by FFD teams
3. The standardisation of FFD core aims objectives and team composition nationally would benefit service integration
4. Certain elements of FFD team composition and skillset should be site specific e.g., WTE allocation, competency framework
5. FFD team would benefit from a structured clinical governance approach regarding initiation/completion of CGA in the ED and decision making regarding disposition planning
6. FFD team would benefit from a structured operational governance approach regarding onward referral to integrated care services within the community

**8. Evaluation and metrics**

1. Evaluation of FFD is important to enhance patient experience and outcomes
2. Evaluation of FFD is important to enhance staff experience
3. Greater resources, education and training are required to fully enhance understanding and value of FFD evaluation by team members
4. Evaluation of FFD should include process outcomes only e.g., incidence of ED discharge
5. Evaluation of FFD should include both process and clinical outcomes e.g., incidence of 30-day unscheduled ED revisit and an ADL index measure
6. Evaluation of FFD should include a measure of patient experience of FFD
7. FFD team should report quarterly metrics to the HSE Acute Hospitals Division
8. FFD team should report biannual metrics to the HSE Acute Hospitals Division
9. There is a need for an UpToDate feedback system for teams reporting to local governance structures

**9. Research**

1. Research is a key component of the FFD model of care
2. Research is seen as a priority by FFD team
3. Research is valued in the FFD model of care
4. Greater efforts are required to enhance understanding and value of research
5. The FFD team research agenda needs to be framed by NCPOP research strategy
6. A national competency framework is required specific to FFD
7. All FFD team members should have completed postgraduate education in care of the older person
8. FFD team should engage in reporting metrics to national repository (e.g., HSE Acute Hospitals Division)

**10. Additional comments**

**Additional file 4: List of statements based on three consensus criteria**

| **Consensus Criteria** | | | | | |
| --- | --- | --- | --- | --- | --- |
|  | **% scoring 7-9 (≥70)** | **Median 7-9, (IQR < 3)** | **Mean 7-9** | **Rank (Based on Mean)** | **Consensus** |
| **Aims of FFD** | | | | | |
| Reduce length of stay of older adults in the ED | 77.77% | 8(2) | 7.53 | 5 | YES |
| Screen for presence and risk of frailty among older adults who present to the ED | 91.84% | 9(1) | 8.31 | 3 | YES |
| Improve the experience and outcomes of older adults living with frailty who present to the ED | 97.95% | 9(0) | 8.76 | 1 | YES |
| Enable early identification and assertive case management of older adults living with frailty to ensure appropriate plan of care | 95.83% | 9(1) | 8.46 | 2 | YES |
| Promote the age attuning of the ED to meet the needs of older adults living with frailty through bespoke pathways, processes and an  interdisciplinary approach to care | 87.5% | 9(1) | 8.13 | 4 | YES |
| **Objectives of FFD** | | | | | |
| Embed Comprehensive Geriatric Assessment (CGA) in the ED through early assessment and intervention of medical, functional, cognitive, and psychosocial  Abilities | 84.85% | 8(1) | 7.96 | 4 | YES |
| Reduce the time from arrival to the ED to initial medical assessment and work-up for older adults living with frailty | 77.78% | 8(2) | 7.47 | 8 | YES |
| Facilitate timely and supported patient discharge from the ED through initiation of referrals to appropriate community-based integrated care services | 95.56% | 9(1) | 8.36 | 2 | YES |
| Reduce the incidence of admission to hospital among older adults living with frailty who present to the ED | 79.99% | 8(2) | 7.80 | 5 | YES |
| Actively support patient advocacy ensuring older adults living with frailty are admitted to an appropriate in-patient setting | 88.89% | 8(2) | 7.80 | 6 | YES |
| Reduce the length of stay for older adults living with frailty admitted to hospital following ED attendance | 77.78% | 8(2) | 7.53 | 7 | YES |
| Reduce incidence of long-term care/nursing home admission following ED attendance | 51.11% | 7(4) | 6.53 | 9 | NO |
| Promote a culture of proactive case management of older adults living with frailty in the ED, ensuring clear communication on the outcome of assessments | 86.67% | 9(1) | 8.24 | 2 | YES |
| Increase awareness of frailty and its presentations in the ED | 93.33 | 9(1) | 8.38 | 1 | YES |
| **Target population** | | | | | |
| Older adults who present to the ED with a fall as their index complaint | 79.55% | 8.5(2) | 7.73 | 6 | YES |
| Older adults who present to the ED with mobility or functional deficits | 88.64% | 8.5(2) | 8.02 | 3 | YES |
| Older adults on multiple medications perceived as vulnerable to side effects | 88.63% | 8(2) | 7.82 | 5 | YES |
| Older adults with cognitive impairment/delirium or a known diagnosis of dementia | 83.72% | 8(1.5) | 7.98 | 4 | YES |
| Older adults who have multiple complex co-morbidities indicative of frailty | 90.90% | 9(1) | 8.30 | 1 | YES |
| All older adults aged ≥75 years who present to the ED during the operational hours of the service | 70.46% | 8(2.25) | 7.05 | 9 | YES |
| All older adults aged ≥65 years who present to the ED during the operational hours of the service | 23.26% | 5(3.5) | 4.65 | 12 | NO |
| All older adults who are identified as frail regardless of triage category | 67.44% | 7(4) | 6.81 | 11 | NO |
| FFD team should use the results of frailty screening to assist with refining their target population for assessment and intervention in the ED | 72.71% | 8(3) | 7.43 | 8 | YES |
| Incremental ˜assessment” from universal use of screening by all ED workers to full assessment by FFD teams | 65.91% | 7.5(3) | 7.00 | 10 | NO |
| FFD team should increase the operational hours of the service to ensure optimised and consistent provision of care | 79.07% | 8(2) | 7.63 | 7 | YES |
| FFD team should operationalise a triage system and assessment area within the ED that is dedicated to frail older adults | 86.36% | 9(1.25) | 8.09 | 2 | YES |
| **Screening and assessment** | | | | | |
| Screening for frailty should commence at triage and a positive frailty screen should signal the need for review by the FFD team | 86.04% | 9(1) | 8.05 | 4 | YES |
| FFD and ED team should commence early assessment after illness acuity has been determined at triage | 90.7% | 9(1) | 8.23 | 3 | YES |
| FFD team should commence a CGA to incorporate a standardised biopsychosocial assessment of frailty, co-morbidity, polypharmacy, cognition, function and mobility, continence, nutrition, psychological and social status | 93.02% | 9(1) | 8.37 | 1 | YES |
| FFD team should use an interdisciplinary assessment proforma as the basis to inform the intervention plan | 93.03% | 9(1) | 8.35 | 2 | YES |
| **Interventions** | | | | | |
| Assertive case management within the ED | 92.85% | 9(1) | 8.29 | 6 | YES |
| Provide patient and family information on the outcome of CGA and ED discharge plan | 90.7% | 9(1) | 8.35 | 4 | YES |
| Complete timely handover to Community Intervention Team/ Community Specialist Teams for Older Persons (CST OP) / Community Health Network following ED discharge | 95.34% | 9(1) | 8.49 | 3 | YES |
| Provide in-reach to in-patient wards following admission to hospital from the ED | 65.11% | 7(4) | 6.65 | 13 | NO |
| Provide patient and family with information on resources and supports available in the community | 88.37% | 9(1) | 8.23 | 8 | YES |
| Prescribe enabling equipment to address unresolved functional deficits, as appropriate | 86.05% | 9(2) | 8.12 | 12 | YES |
| Conduct medicine reconciliation, as appropriate | 95.34% | 9(1) | 8.35 | 5 | YES |
| Provide home exercise/activity programmes, as appropriate | 88.37% | 9(1) | 8.16 | 10 | YES |
| Provide family and/or caregiver education on delirium risk reduction strategies, as appropriate | 95.35% | 9(1) | 8.56 | 1 | YES |
| Educate the patient and family on self-management strategies, as appropriate | 95.35% | 9(1) | 8.51 | 2 | YES |
| Provide nutritional advice, as appropriate | 86.05% | 9(1) | 8.21 | 9 | YES |
| Refer patients with continence issues to follow up services in the community | 90.70% | 9(1) | 8.23 | 7 | YES |
| Engage in person-centred care planning inclusive of initiation of out-of-hospital referral pathways such as CST OP | 90.70% | 9(1) | 8.16 | 11 | YES |
| **Technology** | | | | | |
| Current technology effectively supports FFD | 7.69% | 3(3) | 3.67 | 4 | NO |
| Greater ICT resources are required to fully support FFD | 92.86% | 9(0) | 8.62 | 1 | YES |
| Specific resources to support FFD should include electronic referral pathways to CST OP | 95.24% | 9(1) | 8.50 | 2 | YES |
| Specific resources to support FFD should include a shared e-proforma across primary and secondary care | 81.48% | 9(1) | 8.24 | 3 | YES |
| **Integration of care** | | | | | |
| The development of shared protocols for the FFD model of care nationally would be beneficial in service development and patient care | 83.32% | 9(2) | 8.00 | 1 | YES |
| A national assessment proforma should be developed and implemented for use by FFD teams | 69.04% | 8.5(3) | 7.43 | 6 | NO |
| The standardisation of FFD core aims objectives and team composition nationally would benefit service integration | 85.71% | 9(2) | 7.90 | 2 | YES |
| Certain elements of FFD team composition and skillset should be site specific e.g., WTE allocation, competency framework | 78.57% | 9(2) | 7.62 | 4 | YES |
| FFD team would benefit from a structured clinical governance approach regarding initiation/completion of CGA in the ED and decision making regarding disposition planning | 78.05% | 8(2) | 7.71 | 3 | YES |
| FFD team would benefit from a more structured operational governance approach regarding onward referral to integrated care services within the community | 71.43% | 8(3) | 7.62 | 5 | YES |
| **Evaluation and metrics** | | | | | |
| Evaluation of FFD is important to enhance patient experience and outcomes | 95.12% | 9(1) | 8.44 | 1 | YES |
| Evaluation of FFD is important to enhance staff experience | 90.25% | 9(1) | 8.15 | 3 | YES |
| Greater resources and training are required to fully enhance understanding and value of FFD evaluation by team members | 87.8% | 8(2) | 7.85 | 4 | YES |
| Evaluation of FFD should include process outcomes only e.g., incidence of ED discharge | 38.46% | 5(5.50) | 5.46 | 9 | NO |
| Evaluation of FFD should include both process and clinical outcomes e.g., incidence of 30-day unscheduled ED revisit and an ADL index measure | 80.49% | 8(2) | 7.80 | 5 | YES |
| Evaluation of FFD should include a measure of patient experience of FFD | 90.25% | 9(1) | 8.29 | 2 | YES |
| FFD team should report quarterly metrics to the HSE Acute Hospitals Division | 63.41% | 7(4) | 6.78 | 7 | NO |
| FFD team should report biannual metrics to the HSE Acute Hospitals Division | 57.50% | 7(3.25) | 6.75 | 8 | NO |
| There is a need for an UpToDate feedback system for teams reporting to local governance structures | 78.04% | 8(2) | 7.58 | 6 | YES |
| **Research** | | | | | |
| Is a key component of the FFD model of care | 65.85% | 8(2) | 7.80 | 2 | YES |
| Is seen as a priority by FFD team | 47.5% | 6(3.25) | 6.18 | 8 | NO |
| Is valued in the FFD model of care | 62.5% | 8(3) | 7.40 | 4 | NO |
| Greater efforts are required to enhance understanding and value of research | 78.04 | 8(2) | 7.63 | 3 | YES |
| The FFD team research agenda needs to be framed by NCPOP research strategy | 75.61% | 8(2) | 7.20 | 6 | YES |
| A national competency framework is required specific to FFD | 87.8% | 8(2) | 7.93 | 1 | YES |
| All FFD team members should have completed postgraduate education in care of the older person | 60.97% | 7(3) | 6.54 | 7 | NO |
| FFD team members should engage in reporting metrics to national repository (e.g., HSE Acute Hospitals Division) | 60.97% | 7(3) | 7.24 | 5 | NO |
|  |  |  |  |  |  |

**Additional file 5: Final list of consensus statements based on strictest criteria**

| **Consensus Criteria** | | | |
| --- | --- | --- | --- |
|  | **Median 7-9, (IQR < 3)** | **Rank (Based on Mean)** | **Consensus** |
| **Aims of FFD** | | | |
| Improve the experience and outcomes of older adults living with frailty who present to the ED | 9(0) | 1 | YES |
| Promote the age attuning of the ED to meet the needs of older adults living with frailty through bespoke pathways, processes and an interdisciplinary approach to care | 9(1) | 2 | YES |
| **Objectives of FFD** | | | |
| Embed Comprehensive Geriatric Assessment (CGA) in the ED through early assessment and intervention of medical, functional, cognitive, and psychosocial abilities | 8(1) | 2 | YES |
| Facilitate timely and supported patient discharge from the ED through initiation of referrals to appropriate community-based integrated care services | 9(1) | 1 | YES |
| Reduce the length of stay for older adults living with admitted to hospital following ED attendance | 8(2) | 3 | YES |
| Reduce incidence of long-term care/nursing home admission following ED attendance | 7(4) | 4 | NO |
| **Target population** | | | |
| Older adults who have multiple complex co-morbidities indicative of frailty | 9(1) | 1 | YES |
| All older adults aged ≥65 years who present to the ED during the operational hours of the service | 5(3.5) | 5 | NO |
| All older adults who are identified as frail regardless of triage category | 7(4) | 4 | NO |
| FFD team should use the results of frailty screening to assist with refining their target population for assessment and intervention in the ED | 8(3) | 2 | NO |
| Incremental “assessment” from universal use of screening by all ED workers to full assessment by FFD teams | 7.5(3) | 3 | NO |
| **Screening and assessment** | | | |
| FFD team should commence a CGA to incorporate a standardised biopsychosocial assessment of frailty, co-morbidity, polypharmacy, cognition, function and mobility, continence, nutrition, psychological and social status | 9(1) | 1 | YES |
| FFD team should use an interdisciplinary assessment proforma as the basis to inform the intervention plan | 9(1) | 2 | YES |
| **Interventions** | | | |
| Provide patient and family information on the outcome of CGA and ED discharge plan | 9(1) | 4 | YES |
| Complete timely handover to Community Intervention Team/ Community Specialist Teams for Older Persons (CST OP) / Community Health Network following ED discharge | 9(1) | 3 | YES |
| Provide in-reach to in-patient wards following admission to hospital from the ED | 7(4) | 7 | NO |
| Conduct medicine reconciliation, as appropriate | 9(1) | 5 | YES |
| Provide family and/or caregiver education on delirium risk reduction strategies, as appropriate | 9(1) | 1 | YES |
| Educate the patient and family on self-management strategies, as appropriate | 9(1) | 2 | YES |
| Provide nutritional advice, as appropriate | 9(1) | 6 | YES |
| **Technology** | | | |
| Current technology effectively supports FFD | 3(3) | 4 | NO |
| Greater ICT resources are required to fully support FFD | 9(0) | 1 | YES |
| Specific resources to support FFD should include electronic referral pathways to CST OP | 9(1) | 2 | YES |
| Specific resources to support FFD should include a shared e-proforma across primary and secondary care | 9(1) | 3 | YES |
| **Integration of care** | | | |
| The development of shared protocols for the FFD model of care nationally would be beneficial in service development and patient care | 9(2) | 1 | YES |
| A national assessment proforma should be developed and implemented for use by FFD teams | 8.5(3) | 6 | NO |
| The standardisation of FFD core aims objectives and team composition nationally would benefit service integration | 9(2) | 2 | YES |
| Certain elements of FFD team composition and skillset should be site specific e.g. WTE allocation, competency framework | 9(2) | 4 | YES |
| FFD team would benefit from a structured clinical governance approach regarding initiation/completion of CGA in the ED and decision making regarding disposition planning | 8(2) | 3 | YES |
| FFD team would benefit from a more structured operational governance approach regarding onward referral to integrated care services within the community | 8(3) | 5 | YES |
| **Evaluation and metrics** | | | |
| Evaluation of FFD is important to enhance patient experience and outcomes | 9(1) | 1 | YES |
| Evaluation of FFD is important to enhance staff experience | 9(1) | 3 | YES |
| Greater resources and training are required to fully enhance understanding and value of FFD evaluation by team members | 8(2) | 4 | YES |
| Evaluation of FFD should include process outcomes only e.g., incidence of ED discharge | 5(5.50) | 9 | NO |
| Evaluation of FFD should include both process and clinical outcomes e.g., incidence of 30-day unscheduled ED revisit and an ADL index measure | 8(2) | 5 | YES |
| Evaluation of FFD should include a measure of patient experience of FFD | 9(1) | 2 | YES |
| FFD team should report quarterly metrics to the HSE Acute Hospitals Division | 7(4) | 7 | NO |
| FFD team should report biannual metrics to the HSE Acute Hospitals Division | 7(3.25) | 8 | NO |
| There is a need for an UpToDate feedback system for teams reporting to local governance structures | 8(2) | 6 | YES |
| **Research** | | | |
| Is a key component of the FFD model of care | 8(2) | 1 | YES |
| Is seen as a priority by FFD team | 6(3.25) | 6 | NO |
| Is valued in the FFD model of care | 8(3) | 3 | NO |
| Greater efforts are required to enhance understanding and value of research | 8(2) | 2 | YES |
| The FFD team research agenda needs to be framed by NCPOP research strategy | 8(2) | 4 | YES |
| All FFD team members should have completed postgraduate education in care of the older person | 7(3) | 5 | NO |
|  |  |  |  |
